# Minimally Invasive Laser Treatment of Ureterocele

**DOI:** 10.3389/fped.2019.00106

**Published:** 2019-04-08

**Authors:** Paolo Caione, Simona Gerocarni Nappo, Giuseppe Collura, Ennio Matarazzo, Maida Bada, Laura Del Prete, Michele Innocenzi, Ermelinda Mele, Nicola Capozza

**Affiliations:** Division of Pediatric Urology, Department of Surgery, Bambino Gesù Children's Hospital, Research Institute, Rome, Italy

**Keywords:** ureterocele, laser, hydro-ureteronephrosis, endoscopic treatment, minimally invasive treatment

## Abstract

**Introduction:** Ureterocelemay cause severe pyelo-ureteral obstruction with afebrile urinary tract infections in infants and children. Early decompressive treatment is advocated to reduce the risk of related renal and urinary tract damage. Endoscopic techniques of incision have been offered utilizing diathermic electrode. We adopted laser energy to release the obstruction of the ureterocele and reduce the need of further surgery. Our technique is described and results are presented, compared with a group of matched patients treated by diathermic energy.

**Materials and methods:** Decompression was performed by endoscopic multiple punctures at the basis of the ureterocele. Holmium YAG Laser was utilized with 0.5–0.8 joule energy, through 8–9.8F cystoscope under general anesthesia. The control group received ureterocele incision by diathermic energy through pediatric resettoscope. Foley indwelling catheter was removed after 18–24 h. Renal ultrasound was performed at 1, 3, 6, and 12 months follow-up. Voiding cysto-urethrogram and radionuclide renal scan were done at 6–18 months in selected cases. Statistical analysis was utilized for data evaluation.

**Results:** From January 2012 to December 2017, 64 endoscopic procedures were performed: 49 were ectopic and 15 orthotopicureteroceles. Fifty-three were in duplex systems, mostly ectopic. Mean age at endoscopy was 6.3 months (1–168). Immediate decompression of the ureterocele was obtained, but in five cases (8%) a second endoscopic puncture was necessary at 6–18 months follow-up for recurrent dilatation. Urinary tract infections and *de novo* refluxes occurred in 23.4 and 29.7% in the study group, compared to 38.5 and 61.5% in the 26 controls (*p* < *0.05)*. Further surgery was required in 12 patients (18%) at 1–5 years follow-up (10 in ectopic ureteroceles with duplex systems): seven ureteral reimplantation for reflux, five laparoscopic hemy-nephro-ureterectomy. Orthotopic ureteroceceles had better outcome. Secondary surgery was necessary in 13 patients (50.0%) of control group (*p* < *0.05)*.

**Conclusions:** Early endoscopic decompression should be considered first line treatment of obstructing ureterocele in infants and children. Multiple punctures at the basis of the ureterocele, performed by low laser energy, is resulted a really minimally invasive treatment, providing immediate decompression of the upper urinary tract, and reducing the risk of further aggressive surgery.

## Introduction

Ureterocele (UTC), often associated with complete duplicated collecting system, represents uncommon cause of congenital uretero-vesical obstruction, present from early prenatal age. It can produce severe consequences on renal parenchyma and urinary tract in infants and young children (1, 2). Although this urinary abnormality is known from several decades, a variable incidence is reported, from the highest rate of 1/5,000 to 1/12,000, and it is more often found in females in association with duplex system (in 95% of cases) (3, 4). Controversy still now continues in the management of UTC regarding diagnosis and specially the more appropriate treatment.

It is commonly indicated that the goals of urological management of UTC are to relieve renal parenchymal obstruction, prevent urinary tract infections (UTIs), minimize surgical morbidity and number of procedures, decompress hydronephrosis, and finally decrease the development of *de novo* vesico-ureteral reflux (VUR) (5, 6). Different therapeutical options have been proposed and sometimes discouraged long time: open surgical techniques, as ureterocelectomy, and ureteral reimplantation or open/laparoscopic nephrectomy and hemi-nephrectomy have been often offered (1, 7, 8). More recently minimally invasive endoscopic procedures have been proposed to provide early decompression (9, 10). Newborns and infants who present with sepsis secondary to urinary obstruction may require immediate drainage of the kidney, which can be performed by endoscopic incision (10, 11). The technique of endoscopic UTC treatment in not yet well-established: total UTCunroofing, UTC wall resection or section, basis opening wall incision, single, or several punctures of the sac may be accomplished by different methods, as cold knife, diathermic incision, or laser energy (9, 11).

From 2012 year in our department, we started to use laser energy to perform endoscopic decompression of UTC instead of diathermic electrocautery, with very encouraging results. The literature on endoscopic laser incision of UTC in pediatric age is sparse (9, 10). Aim of our study was to define precisely the minimally invasive technique that we adopted. We evaluated the results and compared with our previous experience using diathermic energy.

## Patients and Methods

### Study Population

The research design was conducted as retrospective study during the last 6 years, from January 2012 to December 2017, at the Division of Pediatric Urology of the “Bambino Gesù” Children's Hospital. The Institutional Ethical Committee approved the study. All the children with diagnosis of UTCat ultrasonography (US) were included, if treated by laser energy through endoscopic access. Both intravesical and extravesical UTCs were considered. Exclusion criteria were UTCs treated differently from endoscopic decompression and patients with comorbidities that may affect the outcome of the treatment. Extracorporeal UTCs prolapsing out of the urethral meatus and patients lost to follow-up were also excluded.

Preoperative diagnostic work-up included renal and urinary tract US scan, voiding cystourethrogram (VCUG), and radionuclide renal scintigraphy: 99 mTc diamino-succinil acid (DMSA) for renal parenchymal uptake evaluation and 99 mTc mercaptoacetil-triglicine (MAG-3) renal scan for urinary elimination study. Urinalysis, urine culture, and kidney function tests were also evaluated preoperatively in all patients.

As control group, records of matched age infants and children who received endoscopic UTC decompression by incision or punctures techniques utilizing diathermic electric energy during the 2009–2011 years with the same preoperative and post-operative work-up were retrospectively examined. The outcomes were compared in the two groups. Fisher exact test and *T*-square test were adopted for statistical analysis, assuming *p* < *0.05* as significant.

### Endoscopic Technique

All the patients were under antimicrobial prophylaxis. The endoscopic procedure was carried on under general anesthesia, using a 8–9,8 Ch cystoscope with 30 degrees scope and 5 Ch diameter straight operative channel. Preliminary endoscopic evaluation was performed to classify the UTC as orthotopic (intravesical) or ectopic (extravesical). Moreover, number, position and morphology of the ipsilateral and contralateral ureteral orifices were checked, in order to confirm or exclude the presence of double renal-ureteral system.

**Figure 1:**
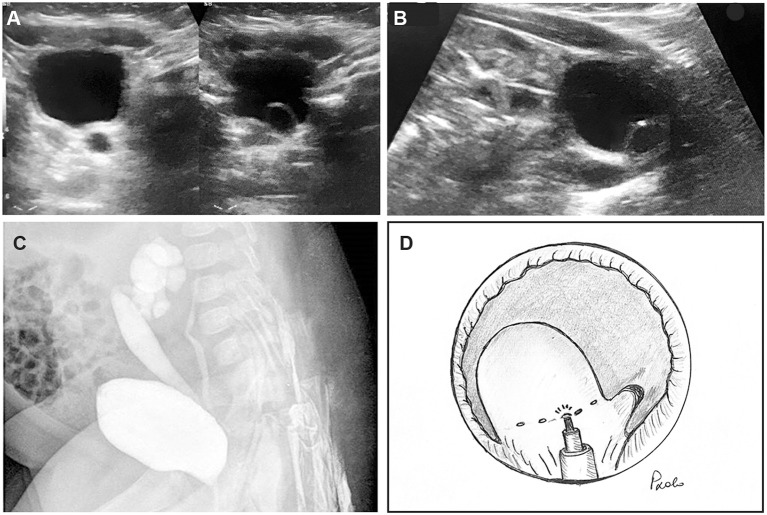
Three-month old female infant with febrile urinary tract infections. **(A)** Ureterocele with dilated ureter was recognized at ultrasonographic scan (US). **(B)** In sagittal plane, ectopic ureterocele in the bladder neck was demonstrated, associated with megaureter. **(C)** Bilateral vesico-ureteral refluxes were found at voiding cysto-urethrogram: grade 4 in the lower ipsilateral pole and grade 2 in the contralateral ureter. **(D)** Diagram of the endoscopic technique of ectopic ureterocele punctures by laser energy: the laser fiber was inserted into a 5F open tip catheter to stabilize it through the operative channel of the cystoscope. A series of 4–8 punctures was accomplished at the basis of the ureterocele.

The source of energy for endoscopic decompression of UTC was holmium: yttrium-aluminum-garnet laser (Holmium:YAG laser) generated by SphinxX Jr 30 W laser machine (Lisa laser products OHG, Katlenburg-Lindau, Germany). We adopted 272 and 550 micron end-finding laser fibers (Quanta System Spa), according to the surgeon preference, with 10–18 W power output. The laser fiber was passed into a 4F open tip ureteral catheter, to better stabilize the probe during the endoscopic procedure. The catheter with the fiber was introduced through the working channel of the cystoscope. As decompressive technique, we adopted to perform multiple (4 to 10) punctures at the UTC basis, close to the bladder neck or trigone, according to the description of Jankowski and Palmer (9) and Timberlake and Corbett (12) (Figures 1A–D). We avoided to create a transverse incision along the distal aspect of the UTC as recommended by Pagano et Al (10) or a “smiling mouth incision” on the UTC wall, as described by Rodriguez (13) and adopted mainly in adult patients (14).The laser setting energy was 0.5–0.8 Joule (mean 0.6 Joule) and frequency pulse rate 5–9 Hz (mean 7 Hz). The bladder was partially filled at low pressure (about 30% of the maximum capacity) to avoid that the UTC could collapse backwards. A 8F Foley catheter was left transurethrally for 18–36 h, so that the balloon could guarantee the UTC walls to collapse, avoiding acute bladder outlet obstruction.

The control group received UTC incision at its basis close to bladder neck and trigonal wall, utilizing diathermic electric energy through a straight electrode of a 10F pediatric resettoscope (Karl Storz GmbH & Co. KG. Tuttlingen, Germany) with a zero degree lens. The incision was continued until visual decompression was noted. A indwelling urinary catheter was left on post-operative day one or two.

### Follow-Up

**Figure 2:**
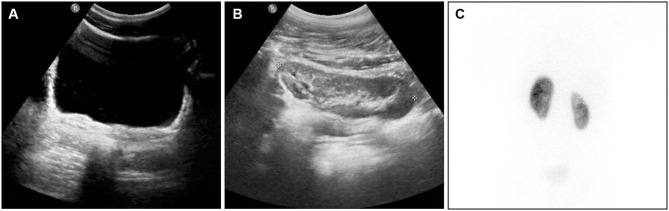
Same patient of Figure 1, at 4 years follow-up from right ureterocele puncture. **(A)** At bladder US scan, the ureterocele wall is hardly visible at the right trigonal angle, acting as flap valve on the ureteral orifice. **(B)** At renal US scan, the upper pole was not dilated, presenting moderate hyper-echogenic changes. **(C)** At DMSA nuclear medicine scan (posterior aspect), the right upper pole is presenting reduced uptake.

Children were discharged within 48 h from surgery, except if severe UTIs or urinary sepsis were present at the hospital admission. All children received antimicrobial prophylaxis postoperatively, that was interrupted according to urinalysis and US results. US check was repeated at 1, 3, 6 months and then yearly. Positive decompression was defined when decreasing of pyelo-ureteral dilatation was observed at US and UTIs or voiding dysfunction were not present (Figures 2A,B). VCUG was done postoperatively only if positive urinalysis and urine culture were found positive for persistent or recurrent UTIs and significant upper tract dilatation was observed without decompressive changes after 6–12 months from endoscopy.

DMSA and MAG-3 renal scan combined with indirect radionuclide voiding cysto-scintigraphy were utilized to check differential renal function with upper pole uptake (Figure 2C). In children who achieved voluntary micturition control indirect radionuclide voiding cysto-scintigraphy was adopted to detect the presence of any VUR.

Indications for further surgery, as re-do endoscopic decompression, open ureterocelectomy with ureteral reimplantation and finally laparoscopic upper pole nephro-ureterectomy, were discussed one by one and carried on according to outcomes and parental preference.

## Results

From January 2012 to December 2017, a total of 69 children presenting obstructive UTC was treated endoscopically by laser multiple punctures. Three patients were lost to follow-up and two had UTC prolapsing outside the external meatus and were excluded. Therefore, the study group was represented by 64 patients: 53 patients (83%) presented renal duplex system (48 females, 90.5% of them). Prenatal diagnosis of upper tract dilatation was present in 43 children (67.2%) and UTC was recognized by fetal US in 18 of them. In 21 children (32.8%) the diagnosis was in postnatal age, from 1 month to 14 years (median 6.3 months), by screening abdominal or renal US in six infants and as consequence of UTIs in 15 children (Table 1).

**Table 1:** Endoscopic decompression of pediatric ureteroceles.

	**Laser punctures**		**Diathermic energy**		***p***
	**Patients**	***%***	**Patients**	***%***	
**DEMOGRAPHY AND CLINICAL PRESENTATIONS**					
Enrolled patients	64	100	26	100	–
Females	55	85.9	21	80.7	n.s.
Double system	53	82.8	22	84.6	n.s.
Double system in females	48	90.5	20	90.4	n.s.
**AGE AT ENDOSCOPY (MONTHS)**					
Range	1–168	–	1–123	–	–
Median	6.3	–	5.9	–	*n.s*.
**PRESENTING SYMPTOMS**					
Prenatal dilatation	43	67.2	15	57.5	n.s.
Post-natal UTIs	15	21.9	8	30.8	<0.05
US screening	6	10.9	2	7.7	n.s.

*UTIs, urinary tract infections; US, ultrasonography*.

Renal agenesis or severe dysplasia was present in two cases with single system UTC. Grade 2–4 VUR was present in 13 out of 29 children who underwent VCUG before endoscopic treatment (44.8%), all in duplex system: four on ipsilateral lower pole, five on contralateral pyelo-ureteral tract. Preoperative renal nuclear medicine study was carries out in 36 children (56%): renal function was decreased at DMSA scan in 21 renal units (58.3%), five out of seven children on single system (mean uptake 38%, in two cases uptake 0–3%) (Table 2). In 23 (79.3%) out of the 29 children with duplex system UTC, the upper pole uptake was reduced (3–15% of total renal uptake).

**Table 2:** Preoperative assessment of laser puncture group of patients.

	**Patients**	***%***
Renal agenesis or severe dysplasia	2	3.12
VUR	8	44.8 (out of 29 VCUG)
DMSA renal scan	36	56
Differential renal function < 45%	21	58.3 (out of 36 DMSA scans)
Orthotopic UTC in single system	5	26.8 (out of 21 with function < 45%)
Ectopic UTC in double system	16	76.2 (out of 21 with function < 45%)

*VUR, vesico-ureteral reflux; VCUG, voiding cystouretrogram; DMSA, diamino-succinil acid; UTC, ureterocele*.

Operative time ranged from 17 to 35 min (median 28 min). Ectopic UTC was recognized in 49 cases (77%), 46 out of the 53 (86.8%) in duplicated ureters (Table 3). Complete or satisfactory immediate decompression of the UTC sac was obtained in all patients at the end of the endoscopic laser procedure. A second multiple punctures endoscopy was necessary in five cases (8%), for recurrent UTC sac bulging and persistent upper tract dilatation (Table 3). In all of them, the punctures were performed using the smaller optic fibers (272 micron).

**Table 3:** Endoscopic treatment of pediatric ureteroceles technique and outcome.

	**Laser punctures**		**Diathermic energy**		***P***
	**Patients**	***%***	**Patients**	***%***	
Total	64	100	26	100	
Orthotopic UTC	15	23	5	19.2	n.s.
Ectopic UTC	49	77	21	80.7	n.s.
Primary decompression	59	92	24	92.3	n.s.
Orthotopic UTC	14	93.3	5	100	n.s.
Ectopic UTC	45	76.3	19	79.2	n.s.
Secondary endoscopic procedure	5	8	2	7.7	n.s.
Orthotopic UTC	0	0	0	0	n.s.
Ectopic UTC	5	100	2	100	n.s.
Febrile urinary infections	15	23.4	10	38.5	<0.05
*De novo* VUR	8^*^	29.7	16	61.5	<0.05
Orthotopic UTC	2^*^	25	5	31.3	<0.05
Ectopic UTC	6^*^	75	11	68.7	<0.05
Further surgery	12	18	13	50.0	<0.05
Vesico-ureteralreimplantation	7	10.9	9	34.6	<0.05
Hemi-nephroureterectomy	5	7.8	4	15.4	<0.05

UTC, Ureterocele; VUR, vesico-ureteral reflux; VCUG, voiding cysto-urethrogram;

* *Out of 27 VCUG*.

No bleeding was observed as consequence of the endo-urological treatment, no significant post-operative pain was described, no other complications occurred as related to the surgical act. All patients voided after postoperative catheter removal. Significant reduction of the upper tract dilatation was observed at US check performed after 3–12 months from the treatment in 59 patients (92%). In 11 cases, complete resolution of the dilatation was achieved at 3–24 months follow-up. Febrile UTIs episodes were observed in 15 children, mostly associated with VUR.

Vesico-ureteral reflux (VUR) was detected by VCUG at 3–16 months from endoscopy (mean 9 months) in eight out of the 27 patients (29.7%) who underwent the diagnostic evaluation for UTIs or for persistent upper tract dilatation: all of them were observed in duplex system. In four cases VUR was present on the upper pole with previously treated UTC, in two cases on the lower pole, in one case on both pyelo-ureteral systems and in the last child on the opposite kidney.

In 12 patients (18%), further surgery was required at 5 years follow-up: 7 vesico-ureteral reimplantation for gross VUR and five laparoscopic upper pole nephro-uretectomy for symptomatic dysplastic hydronephrotic upper renal moiety (Table 3). All of them but one, who received nephrectomy, were in duplex systems.

In the control group, 26 patients (21 females) were treated by diathermic energy: 22 of them (84.6%) presented double system. Age at endoscopy ranged from 1 to 123 months (median 5.0 months). Sex ratio and age at surgery were cross matched with the study group. Demographic and clinical presentation data are described in Table 1 and were found not significantly different from the laser group.

Orthotopic and ectopic UTCs were, respectively 19.2 and 80.7% out of the 26 patients of the control group, with similar proportion observed in the study group (Table 3). The operative time ranged from 16 to 37 min (median 22 min). Primary decompression was achieved in 24 UTCs (92.3%) and a second endoscopic procedure was necessary in two patients. All these results were not significantly different from the study group. In both groups orthotopic UTCs had better outcome, compared to ectopic (Table 3).

Conversely, 10 febrile UTIs (38.5%) and 16 *de novo* developed VUR (61.5%), mostly on the upper pole ureter, were observed during the 5-year follow-up. The difference with the study group was significant. Finally, further surgery was required in 13 patients (50.0%): 9 vesico-ureteral reimplantations (34.6%) and 4 hemi-nephroureterectomies (15.4%). Secondary surgical procedures resulted significantly higher in the control group than in the laser group (*p* < 0.05).

## Discussion

UTC is usually defined as a cystic dilatation of the terminal portion of the ureter inside the bladder basis (1, 2). Depending to the position on the bladder, UTCs are classified as orthotopic or intravesical and ectopic or extravesical. The orthotopic UTC is completely located in the bladder at the trigone angle, mostly combined with a single pyelo-ureteral system. It is more commonly observed in older children and adults (4, 15). The UTC is defined ectopic or extravesical if any portion extends into the bladder neck or urethra (4). The ectopic UTC is the most common presentation, recognized in more than 80% of all of them (15). In our series, we had 77% of extravesical UTCs, diagnosed at cystoscopy. In duplex systems, UTC is related to the upper pole ureter.

The ureter corresponding to the lower pole moiety is often raised and frequently compressed by the UTC, leading to an obstructive megaureter. In other situations, the UTC may distort and bend the lower pole ureteral orifice and/or the contralateral ureteral orifice, leading to VUR. VUR is reported as associated to UTC in duplex systems in 50% on the ipsilateral side and on < 20% on the contralateral side (4–6).Sometimes UTC can be prolapsing into the urethra in the female newborn or infant (2, 3).It is four to seven times more frequent in female sex. In 80% of cases, UTC is related to the upper pole ureter of a duplicated system and it is related to a single system in 20%. In our series, duplex system was present in 82.8% of patients born with UTC (Table 1). It is recognized bilaterally in 10% of them (3, 4, 15).

The UTC is usually obstructing the related upper tract, as it interferes with the urine outlet. Often severe dilatation of the corresponding pyelo-ureteral system is present from prenatal age and nowadays accurate fetal US is able to suspect UTC in a large number of cases (15, 16). *In utero* decompression of prenatally detected has been recently reported in few cases. Laser energy was used by fetoscopic suprapubic cystoscopy, with good results (16–18). In our experience, prenatal diagnosis was positive for fetal hydro-ureteronephrosis in 67.2% of children and was able to predict UTC in about 30% of them (Table 1).

Prenatal diagnosis allows newborns and infants to start proper treatment shortly after birth, avoiding the risk of severe UTIs, or urinary sepsis (7, 10). The choice of the more appropriate therapeutic modality depends on the following criteria: clinical patient's status (mainly febrile UTI or urinary sepsis), patient age, function of the upper pole, refluxing or obstructed ureter, bladder neck obstruction caused by the UTC, position of the UTC (intravesical or ectopic), and finally patients/parents and surgeon's preferences. Different surgical options have been advocated for treatment of obstructive UTC in infants and children. Open surgery included trans-vesical ureterocelectomy and ureteral reimplantation (6, 8). Upper pole partial nephro-ureterectomy in duplex system or complete nephro-ureterectomy in single system can be proposed in case of poor functioning corresponding renal parenchyma (4, 8). Ablative renal surgery is recently offered by different laparoscopic approaches: trans-peritoneal, retroperitoneal via lateral, or posterior access (19).

Endoscopic decompression of the ureterocelic sac and of upper tract dilatation included several modalities, that have been offered along the last two decades: unroofing, incision, and punctures are different methods proposed and adopted to decompress the UTC (20–22). The energy source used for decompression includes Collins knife, diathermic electrocautery, and more recently holmium laser (14, 22). No consensus is present in literature regarding the best endoscopic technique, as punctures or incision, position and size of the opening, as well as utilized instrumentation (15, 20). No randomized controlled studies have been published till now comparing the different endoscopic approaches. Moreover, precise statistical analysis between the different methods is not usually feasible (23). Endoscopic treatment of UTC gained popularity for the easier technique and minimally morbidity compared with open surgery, so that endoscopic procedures have raised as first-line therapy at several centers. As far as historical aspects, Monfort proposed in 1985 to perform a small incision at the UTC basis, instead of the common practice of UTC unroofing (22). The technical refinement of multiple UTC punctures was introduced in 1999 (22). Ben Meir et al. assessed that similar good results were achieved by adopting different technical options: endoscopic puncture or UTC incision, performed by cold knife, or electric diathermy (20). Renal function and bladder or trigone anatomical characteristics were considered as significant issues for UTC endoscopic decompression outcome and for post-operative VUR development in this cohort of patients (20).

The holmium laser has been demonstrated as a very effective and good handling source of energy in endo-urology, not only for stones fragmentation but also for tissues cutting and ablation (24, 25). Laser energy is able to decompress thin and thick UTCs, varying the energy and the frequency: In our experience, we adopted 0.5–0.8 Joule energy with 5–9 Hz frequency, depending to the quality and thickness of the UTC wall. The laser energy has the property to ablate or vaporize the tissues with fine precision and with less surrounding cellular damage or thermic effects than diathermic electrocautery. Thus, the probability of incision scarring and resealing is less evident than that observed in conventional incisions or by electrocautery (21).

We adopted in our series the technique of multiple (4–10) thin punctures at the UTC basis, performed by the 272 or 550-micron fibers, depending on the surgeon's preference and on the thickness of the UTC wall. Using the holmium laser energy, we avoided to make a “smiling mouth” incision as described by Rodriguez (13) and Shah et al. (14), technique that we adopted in our previous experience with the use of the diathermic electrocautery incision technique. The laser technique provides very small orifices on the UTC wall, that we consider as safer to avoid the risk of UTC recurrence. The position of the punctures, low at the UTC basis and close to the trigonal or bladder neck wall, minimizes the risk of secondary VUR into the ureter, because the collapsed UTC wall acts as antireflux flap valve mechanism (14). This effect is shown in Figure 2.

We compared the results of the study series with our previous experience, in which we adopted the technique of diathermic cautery incision of the UTC basis till the 2011 year. We reviewed 26 children with UTC treated during the 2009–2011 years by the same surgeons and found *de novo* VUR development in 61.5% of patients instead of 29.7% observed in the laser energy punctures group: the difference was significant (*p* < 0.05). The need of secondary decompression by re-do endoscopy for recurrent obstruction was similar in the laser group of patients (8%), compared with the diathermic group (7.7%) (Table 3). The recurrence of obstruction in the five cases of our experience in the study group was observed when the smaller fiber (272 micron) was utilized and the UTC wall was described as almost thick. In our procedure, we often adopted to use an open tip 4F catheter to stabilize the thin laser fiber within the operative channel of the cystoscope, to guide more accurately the fiber at the UTC basis. Ben Meir et al. and associates described a similar technique in children (20). The need of further open surgery was 18% (12 patients) in the laser group and in 50.0% (13 patients) in the diathermic group. The results were better in single system and orthotopic UTCs, compared with ectopic UTCs associated with double system (Table 3). The difference was consistent with the experience of other Authors (1, 25–28) and it can be explained by the better backing of the bladder wall under the orthotopic UTC that prevents the postoperative VUR onset.

The study presents more than one limitation. A limitation derives from the design characteristics, based on a retrospective analysis of the results from a single center, without synchronous randomization with ahistorical control group. The patients treated by diathermic incision or section were recruited in the 3 years before the study group: the different period of treatment could represent a possible bias on results, as consequence of higher surgical experience in the control group. Moreover, the follow-up period was longer in the diathermic cautery group. Finally, the treatment by holmium laser requires the availability of the specific laser energy equipment, not universally present in all the operating rooms, considering also the related costs.

Conversely, literature is almost sparse on holmium laser punctures for endoscopic decompression in pediatric and neonatal age (9–11, 28). Our study presents the largest number of cases found in literature. Results demonstrated clearly the little invasiveness and the positive results in terms of absence of complications and reduced need of further surgeries in the children by laser punctures, compared with the previously utilized diathermic energy. The technique, although simple and short time consuming, needs nevertheless a precise and accurate fulfillment with an initial mentoring period to achieve the necessary experience.

## Conclusions

The endoscopic technique of multiple punctures at the UTC basis utilizing Holmium laser as energy has been demonstrated as very effective and simple procedure, with short hospital stay, to decompress congenital obstructing UTC in newborns, infants, and children. The multiple puncture technique by laser energy, as we describe in the paper and reported similarly by some Authors (9, 11, 21, 28) should be considered the first line treatment of both, intra-vesical or extra-vesical, UTCs if the laser energy machine is available in the operating room. The need of more invasive laparoscopic or open access surgery has been significantly reduced nowadays in our experience.

## Author Contributions

All authors contributed actively to the study. PC and SGN conceptualized the design of the study. MB, GC, EMa, and MI performed retrospective patients chart review. EMe and NC interpreted data and reviewed literature. PC and NC drafted the manuscript. All authors read, revised, and approved the final manuscript.

### Conflict of Interest Statement

The authors declare that the research was conducted in the absence of any commercial or financial relationships that could be construed as a potential conflict of interest.
